# Red Blood Cell Distribution Width and Neutrophil-to-Lymphocyte Ratio as Markers of Cardiovascular Disease and Vascular Calcification in Chronic Kidney Disease: A Large Cohort Study

**DOI:** 10.3390/metabo16040280

**Published:** 2026-04-20

**Authors:** Anastasios Zagaliotis, Athanasios Roumeliotis, Stefanos Roumeliotis, Ioannis E. Neofytou, Garyfallia Varouktsi, Eirini Leptokaridou-Mourtzila, Aikaterini Stamou, Vasiliki Sgouropoulou, Gordana Kocic, Andrej Veljkovic, Rudolf Bittner, Willi Jahnen-Dechent, Leon J. Schurgers, Vassilios Liakopoulos

**Affiliations:** 1 Second Department of Nephrology, School of Medicine, AHEPA Hospital, Aristotle University of Thessaloniki, 1 St. Kyriakidi Street, 54636 Thessaloniki, Greece; dt.zag6@gmail.com (A.Z.); a_roumeliotis@achilleionmed.gr (A.R.); neo2335@gmail.com (I.E.N.); varoukts@uth.gr (G.V.); eirinileptokaridou@gmail.com (E.L.-M.); katerina_stms@yahoo.gr (A.S.); vsgouro@auth.gr (V.S.); vliak@auth.gr (V.L.); 2 Institute of Biochemistry, Faculty of Medicine, University of Nis, 18108 Nis, Serbia; gordana.kocic@ffns.ac.rs; 3 Serbian Academy of Sciences and Arts, 11000 Beograd, Serbia; 4 Faculty of Medicine, University of Nis, 18000 Nis, Serbia; andrej.veljkovic@medfak.ni.ac.rs; 5 Department of Biochemistry, Cardiovascular Research Institute Maastricht, Maastricht University, 6211 LK Maastricht, The Netherlands; rudolf.bittner@maastrichtuniversity.nl (R.B.); l.schurgers@maastrichtuniversity.nl (L.J.S.); 6 Helmholtz Institute for Biomedical Engineering, Biointerface Lab, RWTH Aachen University Hospital, 52074 Aachen, Germany; willi.jahnen@rwth-aachen.de

**Keywords:** CKD, oxidative stress, fetuin-A, vascular calcification, RDW, NLR, T50, cardiovascular risk factors, cardiovascular disease biomarkers

## Abstract

Background/Objectives: Cardiovascular disease (CVD) in chronic kidney disease (CKD) arises from a multifaceted interplay of pathophysiological processes, including chronic inflammation, oxidative stress (OS), and accelerated vascular calcification (VC). Red blood cell distribution width (RDW) and the neutrophil-to-lymphocyte ratio (NLR) have emerged as simple, inexpensive, and readily available hematological indices that may capture these underlying disturbances. As such, they hold promise as accessible biomarkers for stratifying cardiovascular risk in patients with CKD. Methods: This cross-sectional study enrolled 497 patients, comprising 477 with CKD across all stages and 20 controls. We evaluated the associations of RDW and NLR with both traditional and non-traditional cardiovascular risk factors, as well as with serum calcification propensity (T50). Spearman’s correlation and multivariable regression analysis were used to assess these relationships. Results: Both RDW and NLR were significantly elevated in patients with established CVD (*p* < 0.001 for both) and demonstrated a progressive increase across advancing CKD stages (*p* < 0.001). RDW and NLR showed positive correlations with age, CVD duration, urea, phosphorus, parathormone, CRP, FG23, and mean carotid intima–media thickness (cIMT), while exhibiting inverse correlations with eGFR, serum albumin, hemoglobin, lipids, antioxidants such as superoxide dismutase, fetuin-A, and T50. Additionally, NLR correlated positively with the duration of hypertension and diabetes, as well as with albuminuria. Quartile analysis revealed a stepwise decline in T50 across increasing categories of RDW and NLR, supporting the link with impaired calcification defense. In multivariable analysis, T50 independently predicted NLR (β = −0.013; *p* = 0.018), whereas total cholesterol (β = −0.011; *p* = 0.019) and cIMT (β = 0.38; *p* = 0.018) emerged as independent determinants of RDW. Conclusions: RDW and NLR strongly reflect the burden of inflammation, metabolic disturbance, and vascular dysfunction in patients across the CKD spectrum. The consistent associations with impaired calcification defense and with established cardiovascular risk markers underscore the potential value as accessible indicators of cardiovascular vulnerability in CKD. These findings support incorporating RDW and NLR into routine risk assessment and highlight T50 as a mechanistically relevant determinant of hematologic inflammation profiles.

## 1. Introduction

Cardiovascular disease (CVD) is the leading cause of morbidity and mortality in patients with chronic kidney disease (CKD) [1]. Even in the earliest stages of CKD, CVD is already clinically significant, with patients facing a greater risk of dying from CVD than of progressing to end-stage kidney disease (ESKD) [1,2]. However, this substantial CVD burden remains largely undiagnosed in patients with CKD and cannot be adequately explained by traditional risk factors such as hypertension, diabetes mellitus, obesity, and dyslipidemia. Increasing evidence highlights the pivotal contribution of non-traditional mechanisms, including chronic inflammation, oxidative stress (OS), endothelial dysfunction, vascular calcification (VC), and vascular stiffness (VS), which together drive the accelerated cardiovascular phenotype characteristic of CKD [3,4].

For the early screening and risk stratification of CVD, several surrogate biomarkers have been introduced, including the serum calcification propensity test (T50), which quantifies the serum’s capacity to delay the transition of primary to secondary calciprotein particles. In patients with CKD, lower T50 values are strongly associated with VC, adverse CV outcomes, and increased mortality, underscoring its potential utility as an integrated marker of cardiovascular vulnerability [5,6]. Furthermore, non-invasive vascular imaging techniques, such as carotid intima–media thickness (cIMT) measured by high-resolution ultrasonography, provide valuable clinical insights that complement circulating biomarkers. cIMT is a well-validated indicator of subclinical atherosclerosis and is consistently associated with CV morbidity and mortality, particularly pronounced in individuals with CKD [7,8]. Although these markers offer valuable insights into VC and CV risk, their routine us in everyday clinical practice remains limited. This is largely due to the high cost of the equipment needed, the need for trained personnel to perform and interpret the tests, and the time-consuming nature of these assessments. There is a clear clinical need for simple, inexpensive, and easily measurable biomarkers whose results can be rapidly obtained and immediately interpreted to guide patient care. These criteria may be met by hematological indices derived from routinely performed complete blood counts, such as red blood cell distribution width (RDW) and the neutrophil-to-lymphocyte ratio (NLR). Traditionally, RDW has been used in the differential diagnosis of anemia as a measure of red blood cell size variability, whereas NLR serves as a sensitive marker of systemic inflammation and aids in distinguishing infectious conditions. Increasing evidence, however, indicates that both RDW and NLR extend far beyond these classical roles. They have been associated with VC and CVD, and a strong relationship between RDW and adverse cardiovascular outcomes, including vascular calcification, has been consistently reported. In CKD populations, RDW correlates with C-reactive protein (CRP), central diastolic blood pressure and dephosphorylated–uncarboxylated Matrix Gla Protein, a potent inhibitor of VC [9]. Moreover, RDW has been identified as an independent predictor of cIMT and endothelial dysfunction, and has been linked to several adverse outcomes, including all-cause and CV mortality, as well as progression to ESKD [10]. NLR, in turn, correlates with kidney function, CRP, the duration of CVD, central diastolic blood pressure and circulating dp-ucMGP. In this cohort, dp-ucMGP independently predicted both RDW and NLR, suggesting that these hematological markers may, at least in part, reflect the underlying burden of VC in CKD [9].

In particular, RDW, which is typically elevated in both CVD and CKD [11,12], has repeatedly been identified as an independent predictor of CV mortality and VC in CKD populations [4,13,14]. Large epidemiologic studies further demonstrate that RDW reliably predicts mortality, at least among ESKD patients undergoing hemodialysis (HD) [15]. Similarly, NLR has been proposed as an informative marker of CVD, VC, and systemic inflammation, especially in CKD and ESKD cohorts [16]. Despite these promising associations, the clinical utility of RDW and NLR as routine biomarkers remains under investigation, and they have not yet been incorporated into standard clinical practice. The limited evidence evaluating the clinical value of RDW and NLR in CKD underscores the need for validation in larger, well-characterized cohorts. Therefore, this study aimed to evaluate the associations of RDW and NLR with established CV risk factors in a broad cohort of patients with CKD spanning all disease stages, thereby providing a more comprehensive assessment of their potential clinical utility. We hypothesized that a higher RDW and NLR would independently associate with increased cardiovascular risk among patients with CKD.

## 2. Materials and Methods

### 2.1. Study Design

**Figure 1:**
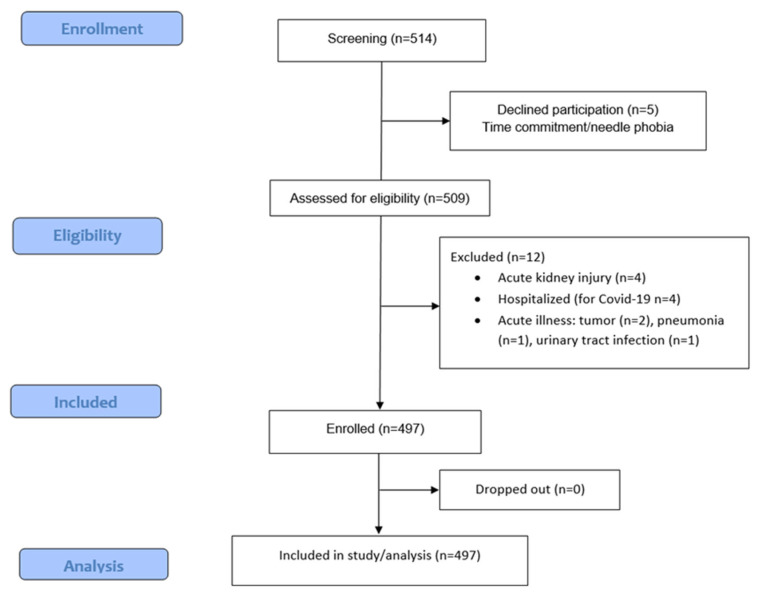
Flowchart enrollment.

In this cross-sectional, single-center study, we recruited 497 patients, with different disease stages of CKD, monitored in our department. Of these, 204 were pre-dialysis outpatients and 293 were ESKD patients. Pre-dialysis CKD individuals were under regular follow-up in the Division of Nephrology and Hypertension at the University General AHEPA Hospital of Thessaloniki (Greece), while dialysis patients underwent maintenance hemodialysis in the Hemodialysis Unit (n = 163) or peritoneal dialysis (PD, n = 130) in the Peritoneal Dialysis Unit of the same center. The period of enrollment was between 1 November 2021 and 1 March 2025. In total 514 patients were screened for participation. Five declined participation and 12 met the exclusion criteria, as detailed in the enrollment flowchart (see Figure 1).

Among pre-dialysis patients, 15 were at Stage 1, 30 at Stage 2, 77 at Stage 3, and 56 at Stage 4, and 6 at Stage 5; the latter had not yet been initiated on any dialysis modality. In addition, 130 patients were on chronic PD, and 163 were on maintenance HD. A total of 20 participants were included as controls (stage 0). Kidney function was assessed with estimated glomerular filtration rate (eGFR), which was calculated using the CKD-EPI 2021 equation. The diagnosis and classification of CKD was based on the National Kidney Foundation Kidney Disease Outcomes Quality Initiative criteria [17].

The exclusion criteria for study participation were the following. Patients who were hospitalized, or suffering from acute kidney injury (AKI), or any other acute medical condition, were not allowed to participate in the study. The definition of AKI was consistent with the Acute Kidney Injury Working Group of KDIGO (Kidney Disease: Improving Global Outcomes) [18]. An acute medical condition was defined as any condition that might affect the markers and the parameters under investigation, such as an infection, a newly diagnosed tumor, or a current hospitalization. At recruitment, we documented demographic, clinical, and laboratory data, including a history of hypertension (HT), type 2 diabetes mellitus (T2DM), and CVD. CVD included coronary artery disease, heart failure (HF), angina, stroke, or peripheral arterial disease. All patients provided informed consent at enrollment. The study was conducted in accordance with the Helsinki Declaration of Human Rights and was approved by the Ethics Committee/Scientific Council of the Medical School of Aristotle University of Thessaloniki (approval no. L235, 14 May 2021).

### 2.2. Laboratory Methods

Blood samples were collected in the morning from all patients after 10–12 h of fasting. In HD patients, samples were taken before the start of a mid-week dialysis session. The parameters measured were creatinine, urea, potassium, sodium, calcium, phosphorus, CRP, parathormone, glycated hemoglobin (HbA1c), triglycerides (TGs), total cholesterol, low-density lipoprotein (LDL), high-density lipoprotein (HDL), serum albumin, urinary-albumin- and protein-to-creatine ratio (UACR and UPCR), and total blood count for white cells, hemoglobin, platelets, and RDW. Furthermore, parameters of the OS were also measured, including malondialdehyde (MDA), advanced oxidation products (AOPPs), fetuin-A, FGF23, catalase, serum xanthine oxidase (XO), and superoxidase dismutase (SOD) activity. Additionally, serum calcification propensity was assessed using the T50 test.

#### 2.2.1. Standard Laboratory Parameters

All routine laboratory parameters were performed within 2 h at the laboratory of the University Hospital AHEPA in Thessaloniki. RDW, neutrophil, and lymphocyte were measured as part of the total blood count using the automatic hematology analyzer Sysmex XE-5000 (Sysmex Corporation, Kobe, Japan). RDW was expressed as RDW-CV (%), as this parameter was consistently available in our dataset and is routinely reported in daily clinical practice, given its widespread use in real-world settings. RDW-SD values were not systematically recorded, so they were not included in the present analysis. The reference range for RDW-CV was 12.0–14.0%. Hereafter, RDW denotes RDW-CV in all instances within the text. Furthermore, the biochemical samples were centrifuged at 2000–3000 rpm, the serum was collected, and sodium, potassium, calcium, phosphate, glucose, creatinine, urea, total cholesterol, LDL, HDL, TGs, and serum albumin were measured.

#### 2.2.2. Oxidative Stress Parameters

For the additional OS indices measurements, the blood samples were centrifuged, divided into aliquots, and stored at −80 °C. Samples were transferred in one batch to the Institute of Biochemistry, Faculty of Medicine in Niš, Serbia. Quality control samples were included in every analytical run. Both inter-assay and intra-assay coefficients of variation kept under 10%. Lipid peroxidation was evaluated by quantifying plasma malondialdehyde (MDA) levels using spectrophotometry. This method relies on the reaction between MDA and thiobarbituric acid (TBA), which produces a colored MDA–TBA complex. MDA concentrations were reported in µM/L [19]. Protein oxidative modification was evaluated through quantifying plasma advanced oxidation protein products (AOPP) levels using spectrophotometry. The results were expressed as chloramine T equivalents. Plasma samples were diluted 1:10 before analysis [20]. Catalase activity, an antioxidant enzyme, was quantified spectrophotometrically using H_2_O_2_ as the substrate. H_2_O_2_ forms a stable yellow complex with molybdate salts. A decrease in absorbance indicates catalase activity, which is expressed in kat/L [21]. Serum xanthine oxidase (XO) activity was measured using a spectrophotometer. This method relies on tracking the release of uric acid from xanthine as the substrate [22]. Minor adjustments to the assay have been made [23]. The result was expressed in U/L. Total superoxidase dismutase (SOD) activity was calculated using Minami and Yoshikawa’s spectrophotometric method, which is based on formazan-colored product formation [24]. The pyrogallol autooxidation-generated superoxide anion (O^2−^) produces a colored product when it reacts with nitro blue tetrazolium. As an O^2−^ scavenger, SOD prevents this reaction. The unit used to express the enzyme activity was U/mL. The supplier of chemicals was Sigma Company (St. Louis, MO, USA) [25]. A human fetuin-A enzyme-linked immunosorbent assay (ELISA) kit was used to measure serum fetuin-A (Epitope Diagnostics Inc., San Diego, CA, USA) that makes use of the two-site “sandwich” method using antibodies that attach to various fetuin-A epitopes. The coefficients of variation for the intra-assay and inter-assay were less than 5.5% and 6.8%, respectively [26].

#### 2.2.3. Serum Calcification Propensity T50 Test

T50 was measured using the T50 calciprotein crystallization test on non-fasting serum samples in 116 samples. Samples were collected and stored at a temperature of −80 °C. Subsequently, they were transferred to Department of Biochemistry, Cardiovascular Research Institute Maastricht, Maastricht University Medical Center, 6200 MD, Maastricht, the Netherlands. T50 was measured through nephelometry as described by Pasch et al. [5]. The assay coefficient of variation amounted to 6%.

### 2.3. Hemodynamic Variables Assessment

We additionally included measurements of parameters indicative of hemodynamic status. Specifically, central aortic blood pressure, diastolic blood pressure (DBP), systolic blood pressure (SBP), carotid–femoral pulse wave velocity (PWV), cardiac rhythm, pulse pressure, and the augmentation index (AI) were measured with the Mobil-O-Graph device (IEM, Stolberg, Germany). Because of its confirmed accuracy in assessing hemodynamic parameters, Mobil-O-Graph was chosen as the instrument of choice. The sphygmomanometer was validated according to the ESH/ESC criteria [27]. Finally, cIMT was measured by ultrasound by an experienced vascular sonographer using a high-resolution linear transducer in B-mode, placed at the common carotid artery 10–20 mm proximally to the bifurcation. A total of three measurements were taken in each carotid for mean calculation.

### 2.4. Statistics

To assess whether the data conformed to a normal distribution, the Kolmogorov–Smirnov test was employed. Normally distributed variables were characterized by their mean and standard deviation, while non-normally distributed variables were described using the median and range. Associations between RDW, NLR, and other variables were evaluated using Spearman’s correlation coefficient. The cohort was divided into four quartiles based on the median values of RDW (14.5%) and NLR (3.2): quartile 1 comprised individuals below both medians; quartile 2 included those with below-median NLR and above-median RDW; quartile 3 consisted of those with above-median NLR and below-median RDW; and quartile 4 encompassed individuals above both medians. Depending on the variable, either the Kruskal–Wallis test or the chi-square test was used to compare groups. To identify the predictors of RDW and NLR, stepwise multiple regression analyses were performed, incorporating variables that were significant in bivariate analyses. Variables that did not follow a normal distribution were log-transformed before regression analysis. All the statistical analyses were conducted using IBM SPSS Statistics 18.0 for Windows, and a *p*-value < 0.05 was considered statistically significant.

## 3. Results

Clinical, hematologic, and biochemical characteristics were stratified according to CKD stage. The study population had a median age of 69 years and consisted predominantly of male participants (62.8%). Hypertension was highly prevalent (74.8%), particularly in CKD stages 3–5, with a median disease duration of 15 years. T2DM was present in 42.3% of the cohort and showed increased prevalence in stage 5, where the median duration was 20 years. A detailed overview of the demographics and baseline is provided in Table 1.

Spearman’s analysis revealed correlations of RDW with various risk factors for CVD in CKD (Table 2). In particular, RDW exhibited a significant positive correlation with NLR, age, duration of CVD, urea, phosphorus, parathormone, CRP, HbA1c, IMT average and FGF23, and negative correlations with mean blood pressure, diastolic blood pressure, heart rate, central diastolic blood pressure, GFR, hemoglobin, sodium, calcium, serum albumin, total cholesterol, HDL, LDL, MDA, SOD activity, fetuin-A and T50.

**Figure 2:**
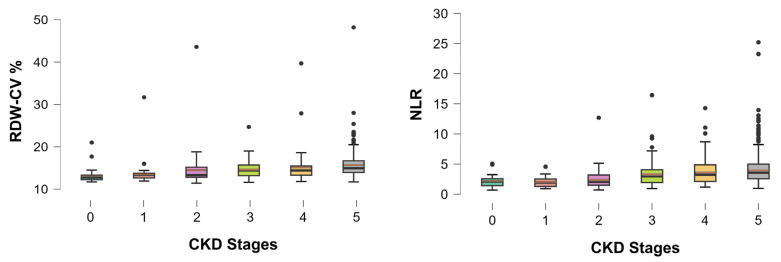
Distribution of RDW and NLR across CKD stages.

NLR was positively correlated with age, the duration of hypertension, the duration of diabetes, the duration of CVD, urea, phosphorus, parathormone, CRP, HbA1c, albuminuria, IMT and FGF23, and was inversely with mean blood pressure, diastolic blood pressure, heart rate, central diastolic blood pressure, GFR, hemoglobin, sodium, calcium, serum albumin, total cholesterol, HDL, LDL, MDA, SOD activity, fetuin-A and T50. Moreover, statistically significant differences in the RDW and NLR values were observed across all stages of CKD (Kruskal–Wallis test, *p* < 0.001 for both; Figure 2), as well as among different causes of nephropathy (patients with cardiorenal syndrome had higher median RDW: 15.75 vs. 14.5, *p* < 0.001, and NLR: 3.74 vs. 3.20, *p* = 0.001). Specifically, RDW and NLR were both gradually increased as CKD progressed and were further exacerbated when entering any dialysis technique. The NLR values were significantly higher in males compared to females [median (range): 3.36 (0.92–25.2) vs. 2.8 (0.7–13.06); *p* = 0.001], whereas RDW did not differ significantly between the sexes, although values tended to be higher in males [median (range): 14.6 (11.7–48.2) vs. 14.4 (11.4–31.7); *p* = 0.056].

Red blood cell distribution width-coefficient variation (RDW-CV) and neutrophil-to-lymphocyte ratio (NLR) values in patients with pre-dialysis stages of CKD (stages 0–5). Boxplots show the interquartile range (Q1 to Q3), with median values indicated by the horizontal black line within each box and the mean values indicated by the brown horizontal line respectively. Individual data points represent outliers. Kruskal–Wallis test: *p* < 0.001 for both RDW and NLR. CKD: chronic kidney disease.

RDW medians (IQR): Stage 1: 11.7% (11.7–21.0); Stage 2: 13.4% (11.9–31.7); Stage 3: 13.3% (11.4–43.6); Stage 4: 14.4% (11.6–24.7); and Stage 5: 14.4% (11.8–39.7).

NLR medians (IQR): Stage 1: 2.1 (0.7–5.1); Stage 2: 2.0 (0.9–4.6); Stage 3: 2.1 (0.7–12.7); Stage 4: 3.0 (0.9–16.5); and Stage 5: 3.3 (1.2–14.3).

**Figure 3:**
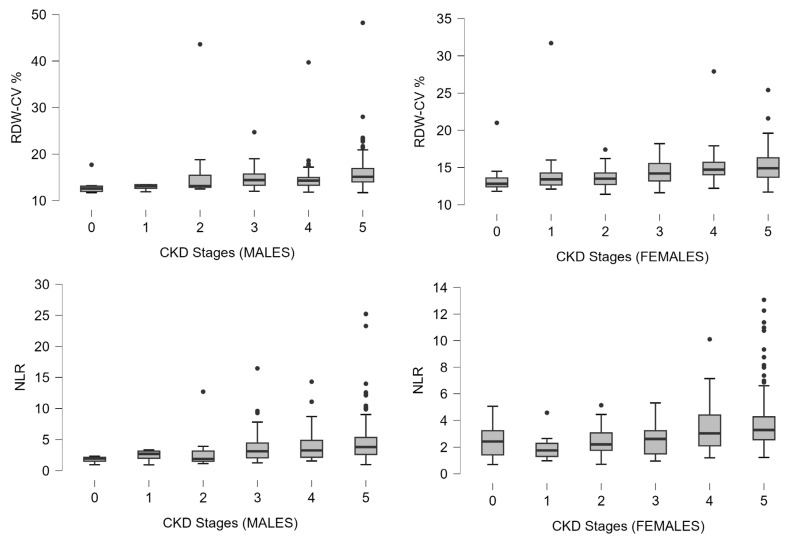
Sex-stratified distribution of RDW and NLR across CKD stages.

Sex-stratified analysis was performed to further explore whether the associations of RDW and NLR with CKD stage, CVD burden, and vascular markers differed between males and females. RDW and NLR were also increased as CKD stage advanced in both sexes, showing a similar pattern to the main analysis (see Figure 2 and Figure 3). Renal disease severity was also assessed using eGFR. In males, RDW and NLR were both inversely associated with eGFR (r = −0.23, *p* < 0.001, and r = 0.24, *p* < 0.001, respectively). Similarly, in females, both RDW and NLR were inversely associated with eGFR (r = −0.34, *p* < 0.001, and r = −0.33, *p* < 0.001, respectively). Regarding CVD, RDW was positively associated with years of CVD in both males (r = 0.21, *p* < 0.001) and females (r = 0.15; *p* = 0.047), whereas NLR showed a significant positive association only in males (r = 0.29; *p* < 0.001) but not in females (*p* = 0.058). For vascular markers, RDW was positively associated with cIMT in both males and females (r = 0.15, *p* = 0.018, and r = 0.18, *p* = 0.026, respectively). NLR was positively associated with cIMT in males (r = 0.16; *p* = 0.016), whereas no significant association was observed in females (*p* = 0.79). With respect to serum calcification propensity, NLR was inversely associated with T50 in both males (r = −0.42; *p* < 0.001) and females (r = −0.23; *p* = 0.014). RDW was inversely associated with T50 in males (r = −0.23; *p* = 0.045), while the corresponding association in females was in the same direction but it did not reach statistical significance (*p* = 0.2).

Distribution of RDW-CV and NLR across CKD stages in males and females. Boxplots show the interquartile range (Q1 to Q3), with median values indicated by the horizontal line within each box. Individual data points represent outliers. Kruskal–Wallis test: *p* < 0.001 for both RDW and NLR. CKD: chronic kidney disease; NLR: neutrophil-to-lymphocyte ratio; and RDW-CV: red cell distribution width—coefficient variation.

Subgroup analyses were performed to assess RDW and NLR values according to the presence of specific comorbidities. In CKD patients with diabetes, both RDW and NLR were significantly higher compared to those without diabetes [median (range): 14.7 (11.7–39.7) vs. 14.4 (11.4–48.2) for RDW, and 3.3 (0.98–25.2) vs. 3.1 (0.7–14.3) for NLR; *p* = 0.03 and *p* = 0.016, respectively], using the Kruskal–Wallis test. Similarly, patients with a positive history of CVD showed higher RDW and NLR values compared to those without (*p* < 0.001 for both). In addition, RDW exhibited higher values in patients with carotid artery disease (CAD) and atheromatic plaques in both carotids (*p* < 0.001). 

We divided our cohort into quartiles according to median RDW (14.5%) and median NLR (3.23). In particular, quartile 1: below-median RDW and below-median NLR; quartile 2: below-median NLR and above-median RDW; quartile 3: above-median NLR and below-median RDW; and quartile 4: above-median NLR and above-median RDW (Table 3).

Urea, PTH, CRP, and FGF23 were progressively increased across quartiles, whereas hemoglobin, serum albumin, and T50 were progressively decreased across quartiles.

Multiple linear regression analysis was performed to identify the independent predictors of NLR and RDW. In the model with NLR as the dependent variable, only T50 emerged as an independent predictor [β = −0.013, 95% CI: (−0.023, −0.002), and *p* = 0.018], after adjustment for potential confounders. Correspondingly, in the model with RDW as the dependent variable, total cholesterol [β = −0.011, 95% CI: (−0.02, −0.002), and *p* = 0.019], and IMT [β = 0.38, 95% CI: (0.067, 0.702), and *p* = 0.018] were independently associated with RDW.

## 4. Discussion

In this cross-sectional study of 497 patients with CKD, we found that both RDW and NLR were gradually increased with the progression of CKD. Although there were no significant differences at early stages of CKD (1 + 2), these markers started to rise significantly at stage 3, increased further in stage 4 and were exacerbated at ESKD. Moreover, this abrupt increase in the fifth stage was independent of imitation of dialysis or dialysis modality. Moreover, these two markers were strongly associated with a broad spectrum of traditional and non-traditional CV risk factors. Both indices were correlated positively with age and the duration of CVD and were significantly elevated in individuals with established CVD, supporting their relevance as markers of CV risk. NLR demonstrated additional associations with duration of hypertension and T2DM, as well as with albuminuria, all recognized contributors to CV risk [28,29,30]. Further reinforcing their clinical relevance utility, RDW was significantly higher among patients with CAD, and both markers were positively correlated with mean cIMT, a validated marker of subclinical atherosclerosis. Interestingly, total cholesterol was negatively correlated with both RDW and NLR. However, this finding likely reflects the widespread use of statins in this population rather than a true inverse biological relationship.

Interestingly, sex-stratified analysis showed that the overall direction of associations was consistent between sexes, especially for eGFR and T50. However, some associations, mainly those involving NLR with cIMT and CVD duration, and RDW with T50, were statistically significant only in males. These differences may indicate sex-related biological or hormonal differences in the relationship between erythrocyte heterogeneity and vascular calcification processes. Alternatively, the lack of significance in females may be influenced by smaller female sample size indicating a reduced statistical power.

The findings of our study align closely with previous research demonstrating strong associations between RDW, NLR and CVD in the general population. In a meta-analysis of older adults, each 1% increment in RDW was associated with a 22% higher risk of all-cause mortality (Hazard Ratio 1.22; 95% CI, 1.15–1.30; and *p* < 0.001) [31]. Similarly, in a cohort of 4111 patients with stable coronary artery disease, Tonelli et al. reported that individuals in the highest RDW quartile had a 34% increased risk of CV events compared with those in the lowest quartile [32]. In chronic HF, Felker et al. showed that each 1% increase in RDW independently predicted a 17% increase in mortality, irrespective of hemoglobin levels and renal function [33]. Parallel evidence supports the prognostic value of NLR. Elevated NLR has consistently predicted worse short- and long-term outcomes in acute coronary syndromes. For example, Tamhane et al. demonstrated that higher admission NLR values were associated with approximately a twofold increase in mortality [34]. Beyond mortality, elevated NLR has also correlated with markers of disease severity, including larger infarct size, lower left ventricular ejection fraction, and increased risk of subsequent heart failure [35,36,37,38]. In CKD and dialysis populations, substantial evidence also supports the prognostic significance of RDW and NLR. Among patients undergoing chronic hemodialysis, Oh et al. showed that elevated RDW independently predicted all-cause mortality, with individuals in the higher RDW categories experiencing approximately a 40% increased risk of death [39]. In a large cohort of more than 100,000 HD patients, Vashistha et al. demonstrated that each 1% increase in RDW was associated with a 19% higher mortality risk [15], even after adjustment for iron status, erythropoiesis-stimulating agent use, and markers of malnutrition and inflammation.

Similarly, multiple studies have demonstrated that RDW is a reliable prognostic indicator of both all-cause and CV mortality in HD patients, independent of anemia status [13,15,40]. Comparable findings have been reported in PD patients, in whom elevated RDW was also associated with increased hospitalization [41,42,43]. Beyond dialysis populations, RDW has also been strongly linked to increased cardiovascular adverse events and mortality in pre-dialysis stages of CKD. In retrospective CKD cohorts, Harder et al. identified elevated RDW as an independent predictor of a greater burden of coronary artery calcification [44], reinforcing its relationship with subclinical atherosclerosis. Further, Kor et al. showed that the combination of abnormal mean corpuscular volume (MCV) and high RDW conferred an even greater mortality risk [45]. Consistent with these findings, Lu et al. reported that higher RDW levels were independently associated with increased adverse CV outcomes in patients with CKD [46]. In agreement with these findings, our earlier prospective study demonstrated that RDW was a significant and independent predictor of all-cause mortality, CV mortality, and CV events in the CKD population with diabetic kidney disease [10].

Accordingly, with respect to NLR, accumulating evidence supports its association with both CVD and renal function impairment in CKD populations. In a large prospective cohort of more than 5000 patients, elevated NLR was associated with higher mortality and more rapid CKD progression [47]. More recently, a 2025 meta-analysis including over 26,000 patients with CKD identified NLR as a significant prognostic marker of renal function decline and reported its association with an increased risk of major adverse CVD events (Odd Ratio, OR: 1.42), cardiovascular mortality (OR: 1.21), and all-cause mortality (OR: 1.22) [48]. Consistent with these findings, our study also demonstrated an inverse relationship between both RDW and NLR and eGFR at baseline. Although this relationship does not establish a causal link between elevated RDW or NLR and accelerated CKD progression, it may indicate that these readily accessible hematologic indices reflect cumulative renal injury. Thus, higher RDW and NLR values could potentially signal greater susceptibility to future kidney function decline. For NLR specifically, previous studies have further demonstrated its association not only with kidney function [49], but also with the rate of progression from stage 4 CKD to dialysis initiation [50]. Moreover, NLR has shown prognostic value in several glomerular diseases, including IgA nephropathy, lupus, and ANCA-associated vasculitis, where elevated levels predicted faster deterioration of kidney function [51,52,53]. Our findings suggest that RDW and NLR are also closely linked to several non-traditional CV risk factors, reflecting the chronic inflammation and uremia-related metabolic disturbances that characterize CKD. Both indices were positively associated with serum albumin, parameters widely recognized as markers of chronic inflammation and metabolically dysregulated CKD [54], all of which have been consistently linked to adverse cardiovascular outcomes [55,56,57].

Beyond inflammatory and metabolic burden, RDW and NLR were also associated with biomarkers reflecting OS and impaired VC defense. Specifically, SOD—a key antioxidant—and fetuin-A—an inhibitor of ectopic calcification—were negatively correlated with both indices. Furthermore, the inverse association between RDW, NLR, and T50 underscores their relationship with reduced serum calcification resistance. Together, these findings indicate that elevated RDW and NLR coincide with a more oxidative, pro-calcific, and biologically hostile milieu. Such an environment is known to contribute to endothelial dysfunction and to accelerate vascular aging, thereby providing a plausible mechanistic link between these hematologic markers and heightened cardiovascular risk in CKD. At the same time, the presence of variability and individual outliers across CKD stages underscores the heterogeneity of these biomarkers within CKD population. This indicates the multifactorial regulation of RDW and NLR, which are influenced not only by renal function but also by inflammation, nutritional status, anemia, and comorbidities.

Building upon these observations, a potential explanation linking RDW with CV risk in CKD relates to alterations in erythrocyte morphology and function. RDW shows anisocytosis, which may arise from chronic inflammation, which drives an increased release of immature reticulocytes into circulation. Moreover, pro-inflammatory cytokines, highly prevalent in CKD, can impair erythropoietin response and disrupt iron homeostasis. Concurrently, OS induces structural damage to erythrocyte membranes through reactive oxygen species, shortening erythrocyte lifespan and provoking ineffective erythropoiesis. These processes reveal a mechanistic link between RDW and systemic inflammation, further supporting its association with NLR, a well-established marker of inflammatory background, and by extension, their association with CVD.

T50 is a functional blood test that quantifies the serum’s intrinsic ability to resist the transformation of primary calciprotein particles into secondary, highly calcifying particles [55]. As an overall measure of calcification propensity, it has gained increasing recognition over the past decade and represents a relevant parameter in our study. Consistent with the broader disturbance of mineral metabolism in CKD, both RDW and NLR were negatively correlated with T50. Importantly, T50 remained an independent predictor of NLR in the multivariable analysis, underscoring the robust and independent association between impaired calcification regulation and increased cardiovascular disease. These findings were further supported by the quartile analysis, which demonstrated a gradual decline in T50 across increasing categories of RDW and NLR. Notably, in the intermediate quartiles, where only one of two markers was elevated, the inverse association with T50 appeared for NLR. This finding may reflect the closer biological relationship of NLR with inflammation-driven calcification processes. Consistent with our results, prior studies have linked elevated NLR to vascular calcification in CKD, Wang et al. reported an association between NLR and coronary artery calcification in CKD stage 3–5 patients [56], while Li et al. found that higher NLR was significantly associated with increased prevalence of cardiac valve calcification in newly diagnosed non-dialysis CKD patients [57]. Similarly, among ESKD patients on dialysis evaluated with computed tomography, those with vascular calcification had significantly higher NLR compared than those without VC [58]. In addition, previous studies indicates that lower T50 values, reflecting increased serum calcification propensity, are independently associated with increased cardiovascular adverse events and all-cause mortality in both CKD and dialysis populations [6,59,60].

Evidence supporting the association of RDW and NLR with CVD also stems from our previous investigations. In an earlier study, we demonstrated that elevated RDW was independently associated with increased cIMT as well as higher CV morbidity and mortality [8]. In a subsequent cohort, the novel biomarker dp-ucMGP was identified as a determinant of both RDW and NLR [9], thereby linking these readily available hematologic indices to CVD and VC. Although dp-ucMGP is not widely used in clinical practice, substantial evidence supports its role as a marker of vitamin K deficiency and its association with VC and CVD [61,62]. The present analysis extends our earlier findings by incorporating a larger and well-characterized CKD cohort, and by employing a more comprehensive assessment of CVD pathophysiology through simultaneous evaluation of OS markers, PWV, cIMT, and T50. Notably, we observed a significant inverse relationship between both RDW and NLR with T50, indicating that higher levels of these hematologic indices coincide with a greater serum calcification propensity. Importantly, in multivariable regression analysis, NLR demonstrated a particularly robust association with T50, with a beta coefficient of −0.013 [95% CI: (−0.023, −0.002), *p* = 0.018]. This finding underscores that elevations in these markers reflect an increased calcification burden and, consequently, a higher CV risk. To our knowledge, this is the first study to directly demonstrate an association between RDW, NLR, and T50 in the CKD population.

The correlations observed in this study were modest in magnitude. However, this is consistent with the multifactorial nature of CKD and CVD, where individual biomarkers typically exhibit limited effect sizes. RDW and NLR should therefore interpreted as complementary indicators rather than standalone diagnostic markers. Moreover, the identification of meaningful thresholds remains an open challenge, and justifies further investigation.

This study has several limitations that warrant consideration. First, its cross-sectional design limits the ability to infer direct causality between cardiovascular risk factors, RDW, and NLR. It may also affect the generalizability of our findings, particularly given the likely ethnically homogeneous composition of the study population. Although multivariable adjustments were applied, the possibility of residual confounding from unmeasured or incomplete variables cannot be excluded. Secondly, the lack of longitudinal cardiovascular outcomes restricts our ability to evaluate the prognostic relevance of the observed associations and establish causal relationships. In addition, it precludes the definition of clinically meaningful threshold values, thereby limiting the immediate applicability of RDW and NLR for risk stratification and therapeutic decision making. Although our cohort was large and well-characterized, it represents a specific clinical setting, and therefore external validation in independent populations is warranted. Future prospective and multicenter studies are needed to confirm the present findings, to better define the prognostic value of RDW and NLR, and to clarify their potential role in predicting CKD progression and CV outcomes. In addition, the absence of RDW-SD data constitutes a limitation, as it may provide additional information regarding erythrocyte heterogeneity, thereby limiting a comparative analysis with RDW-CV. Despite these limitations, the study also possesses notable strengths. The comprehensive characterization of inflammatory-, oxidative stress-, and calcification-related markers provides valuable insight into the complex cardiovascular risk phenotype of CKD. In addition, the substantial sample size, extensive data, and inclusion of multiple vascular and biochemical parameters enhance the statistical robustness of our findings and reinforce the clinical relevance of the analyses performed.

## 5. Conclusions

In conclusion, our study demonstrates that RDW and NLR are not only associated with well-established CV risk factors, but also closely linked to systemic inflammation, OS, and impaired defense against VC in patients with CKD, revealing an interplay between inflammation, OS, and erythrocyte dysfunction. Their strong relationship with T50 further supports their link to dysregulated calcification processes. The key advantage of these markers lies in their practical utility: both RDW and NLR are universally available, cost-effective, and rapidly obtained from routine hematological testing, making them highly suitable for real-world cardiovascular risk stratification across all stages of CKD. However, the absence of clinical outcome data limits their direct prognostic applicability. Nevertheless, larger, multi-ethnic, prospective cohort studies—particularly in early CKD—are necessary to validate their prognostic value and clarify their potential role in shaping targeted preventive interventions.

## Figures and Tables

**Table 1:** Βaseline characteristics of the study population per CKD stage. Results for continuous variables are presented as median (range) or mean (S.D.).

Variable	All Patients (n = 497)	Stage 0 (n = 20)	Stage 1 (n = 15)	Stage 2 (n = 30)	Stage 3 (n = 77)	Stage 4 (n = 56)	Stage 5 (n = 299)
Demographic and clinical characteristics							
Age (years)	69 (21–94)	49.0 (21–67)	62 (29–74)	61 (27–78)	72 (25–89)	74 (30–90)	69 (25–94)
Males (n)	312	7	4	14	58	39	190
BMI (kg/m^2^)	26.4 (16.4–45.1)	26.5 (21.7–35.0)	27.0 (18.7–36.6)	26.5 (20.9–36.9)	27.1 (20.6–45.1)	27.2 (20.2–42.2)	25.8 (16.4–43.8)
HT (n)	372	4	5	20	70	50	223
HT duration (years)	15 (0–45)	2.0 (1.0–2.0)	8.0 (1.0–30.0)	8.0 (1.0–45.0)	11.0 (0.0–37.0)	14.0 (2.0–45.0)	15.0 (0.0–45.0)
T2DM (n)	210	0	1	9	35	29	136
T2DM duration (years)	15 (0–54)	–	20.0	10.0 (1.0–30.0)	15.0 (1.0–30.0)	13.0 (1.0–40.0)	20.0 (0–54.0)
CVD (n)	185	0	1	4	30	18	132
CVD duration (years)	0 (0–31.0)	–	9.0	0 (0–10.0)	3 (0–23.0)	3 (0–25.0)	4 (0–31.0)
Hematological parameters							
RDW (%)	14.5 (11.4–48.2)	11.7 (11.7–21.0)	13.4 (11.9–31.7)	13.3 (11.4–43.6)	14.4 (11.6–24.7)	14.4 (11.8–39.7)	15.0 (11.7–48.2)
Hb (g/dL)	12.0 ± 1.7	13.9 ± 2.1	13.2 ± 1.2	13.2 ± 1.8	13.2 ± 2.2	12.1 ± 1.6	11.7 ± 1.3
WBC (cells/μL)	7360 (2170–20,300)	4350 (4350–14,670)	6690 (4500–9200)	7325 (3520–13,110)	7265 (3890–20,300)	8430 (4280–14,370)	7385 (2170–16,720)
NEUT (cells/μL)	4900 (1000–18,100)	3850 (1500–10,500)	3300 (2100–6300)	4400 (1900–11,300)	5100 (2050–18,100)	5600 (2500–11,300)	4990 (1000–15,600)
LYMPH (cells/μL)	1530 (290–6180)	1990 (770–3930)	2040 (1050–3250)	1960 (890–4070)	1640 (490–3950)	1729 (770–3500)	1375 (290–6180)
NLR	3.2 (0.6–25.2)	2.1 (0.7–5.1)	2.0 (0.9–4.6)	2.1 (0.7–12.7)	3.0 (0.9–16.5)	3.3 (1.2–14.3)	3.6 (1.0–25.2)
Hemodynamic parameters							
SBP (mm Hg)	130 (68–197)	130 (124–141)	118 (107–132)	124 (89–156)	137 (68–190)	143 (72–186)	128 (70–197)
DBP (mm Hg)	80 (36–134)	83.5 (69.0–99.0)	80.0 (70.0–86.0)	77.0 (56.0–100.0)	83.0 (44.0–134.0)	86.0 (36.0–111.0)	79.0 (36.0–131.0)
Mean BP (mm Hg)	103 (52–161)	105 (95–118)	96.0 (88.0–104.0)	100 (0–125)	108 (55–154)	111 (52–143)	101 (52–161)
Peripheral pulse pressure (mm Hg)	49 (16–101)	47.0 (42.0–55.0)	40.0 (34.0–52.0)	48.0 (29.0–74.0)	52.0 (24.0–92.0)	58.0 (27.0–96.0)	49.0 (16.0–101.0)
Cardiac rhythm (bpm)	70 (40–127)	82.0 (63.0–110.0)	65.0 (48.0–74.0)	79.0 (0.0–101.0)	70.0 (55.0–120.0)	73.0 (53.0–110.0)	70.0 (40.0–127.0)
Central SBP (mm Hg)	118 (56–181)	118 (111–133)	107 (99–119)	112 (83–146)	126 (56–170)	130 (63–170)	115 (11–181)
Central DBP (mm Hg)	81 (36–137)	86.0 (71.0–100.0)	80.0 (70.0–87.0)	78.0 (0.0–102.0)	85.0 (42.0–137.0)	87.0 (36.0–111.0)	78.0 (36.0–134.0)
Central pulse pressure (mm Hg)	37 (13–85)	35.0 (26.0–41.0)	30.0 (25.0–38.0)	36.0 (0.0–60.0)	39.0 (14.0–77.0)	41.0 (19.0–69.0)	36.0 (13.0–85.0)
AI (%)	23 (2–70)	20.5 (3.0–46.0)	18.0 (11.0–27.0)	26.0 (0.0–41.0)	22.0 (1.0–48.0)	25.0 (6.0–41.0)	24.0 (0.0–50.0)
PWV (m/s)	8.8 (3.1–23.0)	9.4 (7.3–9.7)	8.6 (8.4–9.3)	8.9 (7.3–10.6)	9.3 (6.8–15.1)	9.1 (5.8–12.1)	8.6 (3.1–23.0)
Renal and biochemical parameters							
eGFR (mL/min/1.73 m^2^)	9.0 (0–140)	108 (100–140)	95.0 (83.0–99.0)	73.0 (44.3–94.6)	39.5 (5.0–68.0)	23.1 (5.0–30.0)	5.0 (0.0–19.0)
Sodium (mmol/L)	138 (123–148)	140 (131–143)	140 (138–143)	140 (132–144)	140 (135–146)	140 (130–148)	137 (123–148)
Potassium (mmol/L)	4.6 (2.9–6.9)	4.5 (3.5–5.1)	4.6 (3.8–5.3)	4.4 (3.0–5.2)	4.6 (3.6–5.5)	4.6 (3.3–6.0)	4.5 (2.9–6.9)
Calcium (mg/dL)	9.1 (6.2–11.3)	9.6 (8.4–10.3)	9.5 (9.0–9.8)	9.3 (7.7–10.2)	9.4 (7.1–10.8)	9.2 (6.2–11.0)	8.8 (6.8–11.3)
Phosphorus (mg/dL)	4.2 (1.7–11.0)	3.6 (2.6–4.7)	3.6 (2.7–4.2)	3.4 (1.7–4.5)	3.6 (2.3–6.0)	4.0 (2.1–6.6)	4.8 (1.9–11.0)
Parathormone (pg/mL)	146 (5–2129)	40.0 (5.0–70.9)	45.1 (19.7–71.0)	40.5 (8.0–149.0)	80.0 (10.0–324.0)	115 (7–414)	225 (5–2129)
Albumin (g/dL)	4.0 (2.1–6.8)	4.5 (2.1–5.0)	4.3 (3.9–4.6)	4.3 (2.6–4.9)	4.3 (2.9–5.0)	4.1 (2.4–4.8)	3.9 (2.4–6.8)
Proteinuria (g/g)	0.25 (0.001–33.0)	0.01 (0.00–7.40)	0.01 (0.01–0.40)	0.12 (0.01–8.20)	0.12 (0.00–33.00)	0.39 (0.00–7.20)	0.50 (0.00–9.42)
Albuminuria (mg/g)	0.11 (0.002–19.3)	0.01 (0.00–7.00)	0.01 (0.01–0.17)	0.06 (0.00–8.20)	0.08 (0.00–19.30)	0.29 (0.01–5.80)	0.20 (0.00–5.12)
Lipids and metabolic profile							
Total cholesterol (mg/dL)	157 (41–391)	208 (144–391)	159 (106–220)	152 (60–278)	160 (75–275)	154 (73–358)	153 (41–381)
Triglycerides (mg/dL)	133 (17–879)	85.0 (61.0–573.0)	91.0 (33.0–190.0)	120 (39–580)	127 (31–402)	136 (37–879)	145 (17–644)
HDL cholesterol (mg/dL)	43 (3–139)	57.0 (34.0–136.0)	55.0 (29.0–98.0)	48.5 (20.0–131.0)	47.0 (3.0–139.0)	40.0 (22.0–80.0)	39.0 (14.0–98.0)
LDL cholesterol (mg/dL)	81 (5–232)	132 (77–232)	72.0 (59.0–168.0)	73.5 (10.0–180.0)	85.0 (18.0–181.0)	84.0 (18.0–185.0)	79.0 (5.0–208.0)
CRP (mg/dL)	0.54 (0.01–77.0)	0.30 (0.16–1.64)	0.40 (0.15–1.63)	0.30 (0.08–7.80)	0.50 (0.10–9.27)	0.92 (0.10–77.00)	0.60 (0.05–42.00)
HbA1c (%)	5.8 (3.0–10.7)	5.0 (4.0–10.5)	5.2 (4.6–6.4)	5.6 (4.5–6.8)	5.9 (4.7–10.7)	6.0 (4.3–10.0)	5.8 (3.0–9.6)
Vascular parameters							
IMT average (mm)	7.8 (4.1–16)	6.3 (4.8–8.5)	7.1 (5.0–8.7)	7.0 (4.7–10.1)	7.3 (4.2–11.5)	8.0 (5.2–10.5)	8.2 (4.2–16.0)

RDW: Red blood cell distribution width; WBC: white blood cell count; NEUT: neutrophils; LYMPH: lymphocytes; NLR: neutrophil-to-lymphocyte ratio; BMI: body mass index; HT: hypertension; T2DM: type 2 diabetes mellitus; CVD: cardiovascular disease; SBP: systolic blood pressure; DBP: diastolic blood pressure; BP: blood pressure; AI: augmentation index; PWV: pulse wave velocity; eGFR: estimated glomerular filtration rate; HDL: high-density lipoprotein; LDL: low-density lipoprotein; CRP: c-reactive protein; HbA1c: glycated hemoglobin A1c; and IMT: intima–media thickness.

**Table 2:** Correlations of RDW and NLR with various variables.

	RDW		NLR	
Parameters	*r*	*p*	*r*	*p*
**Anthropometric and hemodynamic parameters**				
Age (years)	0.20	<0.001	0.16	0.001
Duration of hypertension (years)	0.09	0.06	0.13	0.004
Duration of diabetes (years)	0.08	0.07	0.12	0.008
Duration of CVD (years)	0.20	<0.001	0.26	<0.001
Mean blood pressure (mm Hg)	−0.13	0.008	−0.10	0.044
Diastolic blood pressure (mm Hg)	−0.17	0.001	−0.14	0.004
Central systolic blood pressure (mm Hg)	−0.09	0.07	−0.04	0.47
Central diastolic blood pressure (mm Hg)	−0.15	0.003	−0.13	0.009
Peripheral pulse pressure (mm Hg)	0.01	0.85	−0.12	0.81
Heart rate (beats/min)	−0.10	0.04	−0.12	0.016
**CBC and biochemical parameters**				
Hemoglobin (g/dL)	−0.28	<0.001	−0.28	<0.001
HbA1c (%)	0.13	0.007	0.12	0.026
RDW	-	-	0.32	<0.001
GFR (mL/min/1.73 m^2^)	−0.28	<0.001	−0.28	<0.001
Urea (mg/dL)	0.22	<0.001	0.22	<0.001
Sodium (mmol/L)	−0.12	0.009	−0.22	<0.001
Calcium (mg/dL)	−0.15	0.001	−0.22	<0.001
Serum albumin (g/dL)	−0.20	<0.001	−0.24	<0.001
Total cholesterol (mg/dL)	−0.27	<0.001	−0.12	0.009
HDL cholesterol (mg/dL)	−0.23	<0.001	−0.14	0.002
LDL cholesterol (mg/dL)	−0.23	<0.001	−0.11	0.011
Phosphorus (mg/dL)	0.20	<0.001	0.15	0.001
Parathormone (pg/mL)	0.16	<0.001	0.22	<0.001
CRP (mg/dL)	0.26	<0.001	0.30	<0.001
UACR (g/g)	−0.003	0.959	0.14	0.019
**Vascular, oxidative stress, and calcification markers**				
IMT average (mm)	0.18	<0.001	0.13	0.009
FGF23 (pg/mL)	0.26	0.005	0.25	0.007
MDA (µM/L)	−0.17	<0.001	−0.12	0.013
SOD activity (U/mL)	−0.11	0.024	−0.11	0.024
Fetuin-A (g/L)	−0.12	0.019	−0.15	0.003
T50 (minutes)	−0.22	0.017	−0.40	<0.001
NLR	0.32	<0.001	–	–

Spearman’s rho test correlation. RDW: Red blood cell distribution width; NLR: neutrophil-to-lymphocyte ratio; CVD: cardiovascular disease; GFR: glomerular filtration rate; HDL: high-density lipoprotein; LDL: low-density lipoprotein; CRP: C-reactive protein; HbA1c: glycated hemoglobin A1c; IMT: intima–media thickness; FGF23: fibroblast growth factor 23; MDA: malondialdehyde; SOD: superoxide dismutase; UACR: urinary albumin to creatinine ratio.

**Table 3:** Difference in variables according to quartiles based on median RDW/NLR.

	Quartile 1 NLR ≤ 3.23 and RDW ≤ 14.5	Quartile 2 NLR ≤ 3.23 and RDW > 14.5	Quartile 3 NLR > 3.23 and RDW ≤ 14.5	Quartile 4 NLR > 3.23 and RDW > 14.5	*p*-Value
DBP (mmHg)	82 (36–111)	79 (44–119)	81 (57–120)	78 (38–134)	0.046
HR (beats/min)	75 (54–127)	70 (46–110)	70 (0–120)	69 (40–122)	0.011
eGFR (mL/min/1.73 m^2^)	24.2 (0–140)	7 (0–112)	10.5 (0–113)	5 (0–79)	<0.001
Age (years)	64 (21–93)	72 (25–91)	70 (25–94)	70 (30–90)	<0.001
Hypertension duration	8 (0–40)	10 (0–41)	12 (0–45)	12 (0–45)	0.031
Hb (g/dL)	12.8 (9.9–17.4)	11.9 (9–18.2)	11.8 (8.1–16.7)	11.7 (7.2–18.9)	<0.001
Urea (mg/dL)	60 (16–222)	89 (18–228)	90 (20–240)	101 (23–195)	<0.001
Sodium (mEq/lt)	139 (130–148)	139 (124–148)	138 (128–144)	138 (123–148)	<0.001
Calcium (mg/dL)	9.2 (7.1–11.2)	9.2 (7.4–10.8)	9.1 (6.9–11.3)	8.9 (6.2–10.7)	<0.001
Phosphorus (mg/dL)	3.8 (1.7–8.3)	4.5 (2.1–9.4)	4.1 (1.9–11)	4.4 (2.3–8.9)	0.01
PTH (pg/mL)	103.5 (7.2–2129)	135 (15–778)	150 (5–690)	186 (5–1673)	0.001
Serum albumin (g/dL)	4.2 (2.14–6.8)	4.1 (2.6–4.9)	4 (2.9–5)	3.96 (2.36–5)	<0.001
Total cholesterol (mg/dL)	170 (88–391)	146 (51–275)	163 (92–300)	145 (41–381)	<0.001
HDL (mg/dL)	46 (20–139)	41 (17–78)	43 (19–98)	41 (3–86)	0.017
LDL (mg/dL)	89 (28–232)	75 (5–181)	87 (26–208)	72 (10–185)	0.001
CRP (g/dL)	0.33 (0.1–17.5)	0.6 (0.08–77)	0.63 (0.1–19)	0.87 (0.05–42)	<0.001
cIMT (mm)	7.16 (4.8–14.5)	8.16 (4.16–12)	7.92 (4.16–12.5)	8 (4.16–16)	<0.001
MDA (μM/L)	4.2 (1.7–25.7)	3.1 (1.7–18.9)	3.5 (1.85–11.3)	3.4 (1.6–24.4)	0.001
AOPP (mmol/L)	55.6 (0.23–184.4)	72.3 (0.23–201.8)	75.4 (0.9–239.4)	71.7 (0.17–230)	0.059
SOD activity (U/mL)	8.8 (2–9.7)	8.5 (0.5–9.5)	8.44 (3.9–9.5)	8.42 (2.9–9.5)	0.016
FGF23 (RU/mL)	4.9 (0.4–736)	5.4 (1.5–900)	12.3 (1.75–800)	30.8 (1.05–1000)	0.012
T50 (min)	232 (93–402)	226 (55–307)	176 (21–315)	174 (63–284)	0.001

*p*-values of the Kruskal–Wallis test for differences in variables among groups. DBP: diastolic blood pressure; HR: heart rate; eGFR: estimated glomerular filtration rate; Hb: hemoglobin; PTH: parathormone; HDL: high-density cholesterol; LDL: low-density cholesterol; CRP: c-reactive protein; IMT: carotid intima–media thickness; MDA: malondialdehyde; AOPP: advanced oxidation protein products; SOD: superoxide dismutase; and FGF23: fibroblast growth factor 23.

## Data Availability

The original contributions presented in this study are included in the article. Further inquiries can be directed to the corresponding author.

## Abbreviations

The following abbreviations are used in this manuscript:

AI	Augmentation index
AKI	Acute kidney injury
AOPP	Advanced oxidation protein products (also AOPPs)
BP	Blood pressure
CAD	Carotid artery disease
cIMT	Carotid intima–media thickness (also IMT)
CKD	Chronic kidney disease
CKD-EPI	Chronic Kidney Disease Epidemiology Collaboration
CRP	C-reactive protein
CVD	Cardiovascular disease
DBP	Diastolic blood pressure
eGFR	Estimated glomerular filtration rate
ELISA	Enzyme-linked immunosorbent assay
ESKD	End-stage kidney disease
FGF23	Fibroblast growth factor 23 (also FGF-23)
Hb	Hemoglobin
HbA1c	Glycated hemoglobin A1c
HD	Hemodialysis
HDL	High-density lipoprotein
HT	Hypertension
KDIGO	Kidney Disease Improving Global Outcomes
LDL	Low-density lipoprotein
MDA	Malondialdehyde
NLR	Neutrophil-to-lymphocyte ratio
OS	Oxidative stress
PD	Peritoneal dialysis
PTH	Parathormone
PWV	Pulse wave velocity
RDW	Red blood cell distribution width
SBP	Systolic blood pressure
SOD	Superoxide dismutase
T2DM	Type 2 diabetes mellitus
T50	Serum calcification propensity test
UACR	Urinary albumin to creatinine ratio
VC	Vascular calcification
